# Movement as a Positive Modulator of Aging

**DOI:** 10.3390/ijms22126278

**Published:** 2021-06-11

**Authors:** Marie Bičíková, Ludmila Máčová, Dobroslava Jandová, Zdeněk Třískala, Martin Hill

**Affiliations:** 1 Department of Steroids and Proteofactors, Institute of Endocrinology, Národni 8, 11694 Prague, Czech Republic; mhill@endo.cz; 2 Institute for Postgraduate Medical Education, The Third Faculty of Medicine, Charles University, Ruská 87, 10000 Prague, Czech Republic; Dobroslava.Jandova@seznam.cz; 3 Department of the Czech Inspectorate of Spas and Mineral Springs, Ministry of Health, Palackého Náměstí 4, 12801 Prague, Czech Republic; Zdenek.Triskala@mzcr.cz

**Keywords:** exercises, geroscience, vitamin D, COVID-19, immunity, irisin, stress, brain-derived neurotrophic factor (BDNF)

## Abstract

The aging of human populations, including those in Europe, is an indisputable fact. The challenge for the future is not simply prolonging human life at any cost or by any means but rather extending self-sufficiency and quality of life. Even in the most advanced societies, the eternal questions remain. Who will take care of the older generations? Will adult children’s own circumstances be sufficient to support family members as they age? For a range of complex reasons, including socioeconomic conditions, adult children are often unable or unwilling to assume responsibility for the care of older family members. For this reason, it is imperative that aging adults maintain their independence and self-care for as long as possible. Movement is an important part of self-sufficiency. Moreover, movement has been shown to improve patients’ clinical status. At a time when the coronavirus pandemic is disrupting the world, older people are among the most vulnerable. Our paper explores current knowledge and offers insights into the significant benefits of movement for the elderly, including improved immunity. We discuss the biochemical processes of aging and the counteractive effects of exercise and endogenous substances, such as vitamin D.

## 1. Introduction

It is generally accepted that regular exercise, particularly in old age, is beneficial for both physical and mental health. In contrast to physical activities, where evaluations of the effects of movement include observable benefits, such as improved fitness, evaluations of the effect of movement on mental states often depend on subjective perception. The search for objective parameters is the basis of evidence-based studies. Although it has been observed that exercise can help prevent and reduce the development of various neuropsychiatric diseases, specific evidence remains unclear.

Recently, we have shown that patients experiencing anxiety and affective disorders (such as depression) reported improved feelings of wellbeing across a number of parameters following a month-long stay at a health spa [1]. In this study, we have examined the physiological effects of visiting a spa without use of pharmaceutical or medicinal waters and/or peloids, and instead using only climatotherapy and kinesiotherapy (movement) based on physical activities in fresh air, controlled by a physiotherapist and instructors of computer kinesiology. The therapy was based on physiotherapist-directed group hydrokinesiotherapy sessions in a heated swimming pool with a temperature of 27–29 °C and five sessions per week of group Nordic walking (with poles).

Both subjective and objective results were impressive. The Knobloch questionnaire (N-5 self-judging scale) was applied for the subjective patient evaluation. The objective evaluation was based on computer kinesiological examination and the numeric outputs, and biochemical examination. Among the most significant findings was that serotonin levels in all patients increased by a relative 23%, while the amino acid homocysteine (Hcy) decreased by 17.1%. Results showing circulating levels of 95 steroids indicated adrenal cortex activation. Patients subjectively reported overall improvements in mental wellbeing, and biochemical indicators of immune and anti-autoimmune responses provided objective, quantifiable evidence of the positive effects of movement-based therapies.

In our paper, we aim to provide an overview of the beneficial effects of moderate regular exercise on aging. These anti-aging pleiotropic effects were observed on various levels. For the sake of clarity, these effects have therefore been categorized according to the area of human health they affect.

## 2. Frequent Signs of Aging and Countermeasures

### 2.1. Movement and Musculoskeletal Health

#### 2.1.1. Sarcopenia

Sarcopenia is a loss of muscle mass, strength and function often seen in the elderly population. It has a significant negative impact on the clinical status and quality of life of patients, including impaired mobility and reduced independence. Loss of muscle strength accelerates with age. By the age of 75–85 years, a typical person has lost about 45–50% of the muscle strength and by the age of 85, more than 55%. Sarcopenia is relatively common, affecting 10–50% of people over 60 years of age, and it is a precursor syndrome or the physical manifestation of frailty. The review by Sgrò et al. [2] summarizes three possible interventions for sarcopenia: exercise, proper diet and endocrinological balance. These interventions are interconnected and can have positive effects beyond optimizing musculoskeletal health.

#### 2.1.2. Obesity

It is well established that an important precondition for healthy aging is to avoid or reduce obesity, which is involved in the development of many diseases [3]. In addition to a healthy and varied diet, maintaining a healthy microbiotic environment is essential in preventing the development of metabolic disorders, such as obesity and diabetes [4]. Nondigestible carbohydrates (e.g., fibers and pectin) and polyphenols (flavonoids and nonflavonoids, such as green tea) contribute to maintaining healthy gut microbiota. They help in preventing weight gain through their antioxidant and anti-inflammatory properties [5]. According to recent studies, intestinal microbial dysbiosis may cause neurological disorders, leading to brain dysfunction and the pathogenesis of Alzheimer’s disease (AD) [6].

In a review by Gubert et al. [7], the authors presented evidence on the positive role of exercise in neurodegenerative diseases. Specifically, they highlighted the role of exercise in increasing the diversity of gut microbiota, for butyrate-producing bacteria and enhancing colon health. The authors summarized the results of several preclinical studies showing that exercise increases antioxidant enzymes, anti-inflammatory cytokines and anti-apoptotic proteins in intestinal lymphocytes and decreases proinflammatory cytokines. Individual factors such as diet, age and level of physical activity also determine the composition of gut microbiota which, in turn, synthesize neurotransmitters (such as serotonin, norepinephrine, γ-aminobutyric acid and dopamine) capable of neurologically regulating movement [8].

#### 2.1.3. Reactive Oxygen Species and Antioxidants

Evidence that reactive oxygen species (ROS) may play a crucial role in age-related diseases such as sarcopenia, cerebrovascular and neurodegenerative diseases has been emphasized recently [9]. Another critical factor in healthy aging is the accumulation of senescent cells, resulting in tissue dysfunction and frailty [10]. Among internal stressors inducing cellular senescence is ROS which affects a variety of physiological and pathological processes. Lack of exercise, suboptimal amount and quality of sleep, and poor diet all contribute to the accumulation of ROS [11]. One known nonspecific factor influencing ROS accumulation is amino acid Hcy (see below), which plays a negative role in the development of neurodegenerative diseases prevalent in older age groups [12].

The foundation of a strong immune system is a diet rich in vitamin B and antioxidants. Antioxidants have the capacity to lower Hcy levels [13] and enhance natural antioxidants, such as glutathione [14]. A longitudinal study of Hcy and gait speed in older adults [15] and follow-up study [16] reported evidence that higher levels of Hcy are associated with lower muscle strength in women.

#### 2.1.4. Irisin

Irisin—commonly referred to as the “sport hormone”—has been studied intensively in recent years. It is considered to be one of the myokine, a hormone-like polypeptide, that is released from muscle cells after physical activity and induces oxygen consumption in fat cells as well as thermogenesis [17]. Korta et al. provided a detailed explanation of the activity of irisin [18]. Irisin is often described as acting as a hormone through integrins-transmembrane receptors. One of its essential activities is explained by suppression of proinflammatory cytokines. Aging is often accompanied by pathologies such as asthma, immune dysfunction, obesity and type 2 diabetes associated with chronic inflammation. Reduction of oxidative stress, inflammation and physiological stress through exercise and movement is interestingly attributed to the effect of irisin [19]. Physical activities reduce systemic inflammation and cause the protective effect of irisin in the development of associated diseases. Animal experiments on rodents have shown that irisin positively correlates with the expression of a brain-derived neurotrophic factor (see below) in the hippocampus, where it plays a major role in neurogenesis. Thus, physical activity has a positive effect on the nervous system, resulting in better cognitive functions. A recent review [20] focused on the neuroprotective mechanism of physical activity in human studies. Irisin is secreted from the muscles in response to movement and contributes to the neuroprotective effects against cerebral ischemia. Wang et al. propose that irisin could help in the treatment of memory dysfunction and AD [21].

### 2.2. Movement and Neuropsychiatric Health

#### 2.2.1. Stress and Depression

Aging can be defined as a time-dependent physiological deterioration of functions necessary for survival and fertility. The physical aging process affects each individual in different ways and to varying degrees. In addition to apparent physical changes, there are often equally significant changes in mental health.

The incidence of anxiety and affective disorders increases by up to 6% in individuals over the age of 65. Aging is often accompanied by depression [22]. The significant changes that occur due to aging can cause anxiety, feelings of insecurity, loss of self-esteem and depression [23]. Both high levels of acute stress and persistent, long-term stress have been shown to trigger biological, psychological and behavioral changes that lead to further adverse health consequences, such as oxidative stress, neuroinflammation and dysfunction of the blood–brain barrier [24]. Meanwhile, the health benefits of regular exercise therapy in older adults have been shown by several authors [25] and are often attributed to reduced stress and depression [26].

Currently, the most commonly used antidepressants are selective serotonin reuptake inhibitors, such as fluoxetine. The review by Micheli et al. described how fluoxetine and physical exercise both positively counteract depression symptoms in humans and rodents. The authors explain the similarity in the antidepressant effect of fluoxetine and running in the increase in hippocampal neurogenesis and plasticity, although the gene pathways involved were only partially coincident [27].

The evidence of associations between stress, anxiety, affective disorders and a risk of aging-related changes was summarized in 2019 by Lever-van Milligen et al. [28]. The authors compared results of two different treatment interventions for depressed patients: either antidepressants or running. They reported on the findings from their own study and other studies with comparable effects. Running therapy was shown to have a more beneficial impact on biological aging than the antidepressant medication [29,30]. These interventions probably work through different pathophysiological mechanisms. For example, in a 2014 study of 2848 participants aged 18–65 years, Revész et al. [31] verified the theory that telomere length (TL) can serve as a cellular marker for biological age. Shorter TL may predict an individual’s deteriorating metabolic condition. In a later study (2016), the authors showed that the process of advanced biological aging and its effects on TL and telomere maintenance system are negatively influenced by metabolic and physiological stress [32]. The positive effect of physical activity and exercise on TL and possible underlying mechanisms are discussed in a review by Arsenis et al. [33].

#### 2.2.2. Peroxisome Proliferator-Activated Receptor γ

The peroxisome proliferator-activated receptor γ (PPARγ) is a physiological link between mental function and aging, as it is expressed in the brain regions (e.g., hippocampus) that regulate stress and aging. The role of PPARγ in stress and aging has been demonstrated in studies where both stress and aging were observed to induce neuroinflammation and alteration in neuronal metabolism and activity [34]. Stress or aging could predispose the development of dysfunction of one in the other. Faye et al. summarized the findings of neurological mechanisms of stress resilience in aging people [35]. They pointed out that exercise promotes healthy aging and can serve as a treatment for older people with or without psychiatric illnesses. Several authors (e.g., Saxena) have also reported similar findings [36].

#### 2.2.3. Brain-Derived Neurotrophic Factor

In another review, the authors summarized the effects of resistance exercise and endurance activity on the increase in brain-derived neurotrophic factor (BDNF) expressed in the hippocampus [37]. Therefore, the positive effect of exercises on BDNF production is an example of the neuroprotective role of movement.

BDNF decreases with aging; it is a key molecule involved in plastic changes related to learning and memory [38]. Changes in BDNF expression leading to reduced function are associated with aging, exposure to chronic stress and neurodegenerative disease, such as Alzheimer’s disease. BDNF levels are important for maintaining memory functions, mainly in structures responsible for memory processes, such as the hippocampus and parahippocampal areas. BDNF is synthesized as the precursor pro-BDNF that can be stored either in dendrites or axons [39] and undergoes cleavage intra- or extra-cellularly [40,41] to produce a mature BDNF protein. Decreased BDNF levels are accompanied by negative changes in neurotransmitters [42], neuroplasticity and related proteins [43]. Conversely, decreased BDNF also results in enhanced apoptotic activity [43,44] and dystrophic changes [45]. It is possible to increase BDNF levels through aerobic exercise [46], enriched environments [47] and antidepressants [48].

To date, more than 20,000 papers have been published on the importance of BDNF, its significance and the influence of exercise on its production. Some studies have indicated that aerobic exercise may be useful for preventing age-related hippocampal neurodegenerative disorder [49]. According to Ashdown-Franks [50], exercise may improve neuropsychiatric and cognitive symptoms in people with mental disorders. Recent studies that discuss the importance of BDNF for the treatment and prevention of serious neurological disorders, such as AD and Parkinson diseases (PD) [51,52], have shown that the delivery of exogenous BDNF into the patient’s brain had no therapeutic effect on the disease but releasing BDNF by physical activity was neuroprotective. The effect of BDNF on enhanced neuroplasticity has been confirmed by Müller [53], and Molinari [54] has shown its positive effect on brain aging.

In addition to irisin and BDNF, neurotransmitters such as dopamine, noradrenaline, adrenaline and serotonin are also released during physical exercise and thus, contribute to downregulation of neuroinflammatory cytokines in the hippocampus.

### 2.3. Movement and Immune System

Recently, there has been growing evidence that depression is accompanied by increased levels of proinflammatory cytokines [23,55]. In 2015, an extensive study compared the effects of regular, intense exercise and regular, mild exercise on immunity in older adults and patients with various diseases (including chronic viral infections). The earlier findings showed that regular, intense exercise can actually depress immunity, which may be counteracted by nutritional, pharmacological or behavioral intervention. Conversely, regular, mild exercise can serve a powerful tool to improve immunity and health outcomes in both the elderly and patients with various diseases [56].

Ramírez hypothesized a link between innate immunity and the central nervous system. This new theory suggests that the stimulation of interleukin IL-6 and 1L-1β secretion and other immune response factors may be caused by infection or psychological stress and may lead to depressive illnesses [57]. The hyperactivity of the hypothalamic-pituitary-adrenal (HPA) axis leads to decreased synthesis of serotonin and increased cortisol levels which are characteristic of the development of depressive conditions and causing aseptic inflammation in the central nervous system.

Later, some studies described an initial, partial reduction of immunity (“open window”) after an intensive exercise leading to vulnerability to infections. An extensive review of numerous epidemiological studies [58] refutes the theory of “open window”, showing evidence that physically active lifestyles reduce the incidence of bacterial and viral infections, as well as other diseases. The authors have highlighted the benefits of movement and showed that regular physical activity increases immunity and may limit the effects of or delay immunological aging.

The belief that intense exercise weakens the immune system was refuted in another recent study [59]. Baek et al. demonstrated the effect of exercise on immune system function and the interaction between cytokines and antigens by using an animal model and intramuscular infection with a nematode parasite (*Trichinella spiralis*). Their aim was to investigate whether intense exercise increases the risk of infection and what effect it has on immune system function in muscles. Based on the results, the authors strongly criticized the “open window” theory and showed that regular aerobic exercise actually increases immune system function to prevent and defend against infection.

The benefits of regular exercise have further been demonstrated and explained by Nakajima et al. [60]. According to their findings, regular aerobic exercise increases the levels of methylation of the proinflammatory apoptosis-associated protein caspase gene (ASC). Methylation of ASC modulates IL-1b and IL-18 in elderly leukocytes, thereby contributing to attenuating the age-related increase in proinflammatory cytokines.

#### Neuroinflammation, Neurodegeneration and BBB

Recent genomic and functional studies suggest that immune/inflammation pathways are involved in the pathogenesis of AD [61]. The last-named review discussed anti-inflammatory drug candidates for AD and the necessity of new clinical trials targeting neuroinflammation. The chronic neuroinflammation status compromises the blood–brain barrier (BBB) integrity, increases its permeability and leads to the loss of immunological status of the central nervous system (CNS). The BBB has a role in regulating the movement of the immunocompetent cells in the brain [62]. BBB dysfunction could lead to disturbances in neurotrophic factors and to neurological diseases, such as AD or PD [63]. Małkiewicz et al. summarized the positive effects of exercise on BBB permeability balance of pro- and anti-inflammatory cytokines. The same positive effects of exercise on neuroinflammation, pathogenesis, AD and PD are explained by downregulation of microglia activation [64].

The protection of aging brain and the CNS from neurodegeneration pose a significant challenge [65]. Impaired immunity negatively impacts the pathophysiology of neurodegenerative disorders. In the recent review [66], the authors presented findings to show that impaired immunity leads to the formation of misfolded proteins and that accumulation of misfolded proteins in the brain causes neurodegeneration. The relationship between impaired immunity and neurodegenerative diseases has led to the development of immunotherapeutic strategies and to the study of antiviral agents. While antiviral drugs can have a number of negative side effects, they also have a positive effect on some neurodegenerative diseases. Amantadine is one of the best known antivirals widely used in the treatment of PD [67], as an antidepressant [68] and in tests for the treatment of AD [69].

### 2.4. Movement and Endocrine System

#### Vitamins, Hormones and Homocysteine

Aging is associated with an increased prevalence of endocrine disorders which are mainly limited to disrupted secretion in HPA axis. In addition, aging is often accompanied by a decreased sensitivity of the HPA axis to target hormones and feedback loop mechanisms, as well as a decrease in the sensitivity of target tissues to these hormones. Endocrine changes are accompanied by disorders in glucose homeostasis, the loss of muscle and bone mass, and autoimmune and degenerative diseases often associated with reduced receptor sensitivity.

Ligands of mineralocorticoid receptors (MRs) regulate blood pressure, influencing the balance between electrolytes and water in the kidney. The primary mineralocorticoid is aldosterone, but affinity to nuclear MRs is also shown by its precursors deoxycorticosterone, corticosterone and the primary human glucocorticoid cortisol. Nuclear MRs are also expressed in extra adrenal tissues, such as the heart, vascular tissue and adipose tissue. Aging leads to the activation of nuclear MRs, which act profibrotically, leading to vasoconstriction, lowered arterial elasticity and, subsequently, to higher blood pressure. These bioactive steroids are immunosuppressive and anti-inflammatory mediators. Adrenal androgen secretion of aldosterone and renal secretion of renin decrease with age [70].

Elevated blood Hcy levels are currently considered a risk factor for vascular and coronary heart diseases, and negatively impacting nervous system function [71]. Several recent studies report on the positive effects of Nordic walking, underlining its impact on reduced Hcy levels and ensuring adequate supply of vitamin D for an aging organism (calcidiol is a hormone) [72,73].

Vitamin D is correlated to bone health and skeletal muscle function, since its main role in the body is the regulation of calcium-phosphate homeostasis. Various studies have found positive correlations between vitamin D levels and exercise performance [74]. However, vitamin D levels continuously decrease over a lifetime. The beneficial effects of vitamin D supplementation for people over 65 years of age and dosage recommendations are summarized in the Journal of the American Geriatrics Society [75]. The combination of vitamin D supplementation and outdoor activity in fresh air has been demonstrated to yield positive results (lowered Hcy). Meanwhile, vitamin D supplementation without exercise was shown to be insufficient to lower Hcy levels. Lower levels of vitamin D cause missing vitamin D substrate in subcutaneous tissue in older people [76] and the supplementation of vitamin D is important, not only for myoskeletal health but also as a significant immunomodulator. In addition, vitamin D supplements have also been shown to have a beneficial effect on psychiatric conditions [77]. Hormone replacement therapy and a diet enriched with vitamins and amino acids [78] have been shown to be both preventatively and therapeutically beneficial. A recent review [77] evaluated the effective of vitamin D supplementation combined with resistance exercise training on musculoskeletal health in older adults. In agreement with abovementioned studies, the authors recommend vitamin D supplementation resistance exercise. Further studies with a similar design are needed to confirm the findings on positive effects of combined vitamin D supplementation, and resistance exercises are the next necessary studies with the same study design.

### 2.5. Effect of Exercise on Psychiatric Condition—Own Results

Treatments offered in health spas also include supervised physiotherapy, in which the above-described endogenous processes can be activated through movement. For example, prescribed physiotherapy may include activities, such as hydrotherapy, Nordic walking and resistance exercises. As a result of these physical activities, there is often improvement in mental health independent of medication.

A recent study in our laboratory [1] provided evidence of the positive changes in physical and mental wellbeing of psychiatric patients during a month-long stay in a health spa. The patients continued with the same antipsychotic medication protocols as in their home environment, thereby providing a “self-control”. Treatment provided was not based on special diet, drinking therapy or on baths in spa mineral waters, but only on physical activities (see introduction).

In this study, we found statistically significant increases in the levels of 23 steroids, indicating activation of the patients’ adrenal cortex. The positive effects of balneotherapy could be attributed to “hormetic strategy” [79]. As hormetin can be considered a mild stress-induced activation of one or more intracellular pathways of stress response, which in a human organism initiates a series of restorative events in order to counteract, adapt and survive [80]. The other review [81] explains how regular exercise can induce anti-inflammatory and anti-stress responses by immuno-neuroendocrine effects (in persons with deregulated inflammatory system and with stress) by paradoxically reducing the presence of stress hormones and inflammatory cytokines.

## 3. COVID-19 in the Context of Aging

The 2019/2020 coronavirus disease 2019 (COVID-19) pandemic has provided global evidence of the close correlation between overall health status, immunity and aging. It has been observed that one of the key factors in COVID-19 infection, development and mortality is age. Similarly to populations all over the world, the majority (92.8%) of deaths attributed to COVID-19 in Czech Republic occurred in people over 65 years of age [82]. Despite hundreds of studies (over 1,400 published by January 2021) on “COVID-19 and Aging”, it is not yet known exactly why aging has been such a significant risk factor. While it has been observed that many comorbidity indicators (such as obesity, diabetes, hypertension, cardiovascular disease and other significant existing conditions) increase the risk of negative prognosis, we do not yet fully understand why advanced age is an independent factor [83]. The most significant hurdle is, of course, that COVID-19 (and its variants) requires comprehensive research. Therefore, we present only a brief overview of the connection between COVID-19, vitamin D supplementation and movement.

### 3.1. COVID-19 and Aging Immune System

The most significant factor for COVID-19 in older adults is the aging of the immune system. Two basic processes have been observed regarding functional changes to the immune system. The age-associated decline of immune system function—known as immunosenescence and inflammaging—can be attributed to the development of systemic chronic inflammatory environment across the lifespan. Immunosenescence affects innate as well as adaptive immunity, resulting in increased susceptibility to infections, reduced repertoire diversity, thymic involution, insufficient T-cell responses to vaccines and other effects. Meanwhile, inflammaging and chronic low-grade inflammatory status increases sum of inflammatory mediators, IL-6, C-reactive protein, oxidative stress and tissue dysfunction.

Recent data points to both immunosenescence and inflammaging as the key causes of the high mortality rate of older COVID-19 patients. An explanation of the molecular and cellular aspects of this observation is beyond the scope of this current review. However, the involvement of various T-cells, macrophages, cytokines and processes such as proliferation, maturation of immune cells and chemotaxis has been examined elsewhere [84,85]. Some authors suggest that a ‘biological clock’ based on the immune system is more accurate at identifying COVID-19 susceptible individuals than other ‘biological clocks’ [86]. Similarly, sex and age differences in severity of COVID-19 symptoms have been attributed to sex- and age-dependent immune-cell-specific epigenetic and transcriptomic discrepancies [87].

### 3.2. COVID-19 and Vitamin D

There have been numerous studies on the link between COVID-19 and vitamin D—with the vast majority agreeing that vitamin D deficiency is a risk factor. As mentioned earlier, vitamin D levels decrease with increasing age which, in turn, raises the risk factor for COVID-19 in the older adults. The relationship between vitamin D levels and COVID-19 risks can be understood in a number of ways.

Vitamin D helps to regulate the immune system. The regulation of the immune system appears to be intracrine manner dependent on 25-hydroxyvitamin D saturation. In brief, active vitamin D enhances the body’s innate defenses by inducing antimicrobial elements as cathelicidin and defensin, which can block the entry of viruses into cells and suppress their replication. Moreover, it induces viral autophagy and protects against cytokine storms by regulation of proinflammatory cytokines and chemokines release, preventing the overreaction of the adaptive immune response. Modulation of the activity and function of immune cells by vitamin D, especially dendritic cells, macrophages, and T- and B-lymphocytes has an inhibitory and anti-inflammatory effect on adaptive immunity [88].

Although data from available studies can be considered pilot results and caution should be exercised when interpreting the data, there is broad agreement that vitamin D at low doses (1000–2000 IU per day) is safe, not harmful and may potentially prevent a number of acute respiratory infections, including perhaps COVID-19 [89].

### 3.3. Protecting Aging Population against COVID-19

Yang et al. [90] have outlined a protective protocol that can mitigate the COVID-19 risks for older adults. The protocol can be summed up in the triad of: exercise, nutrition and medication. The authors emphasize the positive effects of physical activity on various symptoms accompanying aging and recommend regular exercise, especially walking or running outdoors, for its anti-inflammatory benefits. An unbalanced diet and compromised nutritional status can lead to obesity and disruption of the immune system, including high proinflammatory cytokines production in adipose tissue. Further, obesity often results in physical discomfort and increased reluctance to exercise. The authors also highlight the need for a controlled study to confirm routine use of vitamin D supplementation for older adults at high risk of contracting COVID-19.

## 4. Conclusions

**Figure 1:**
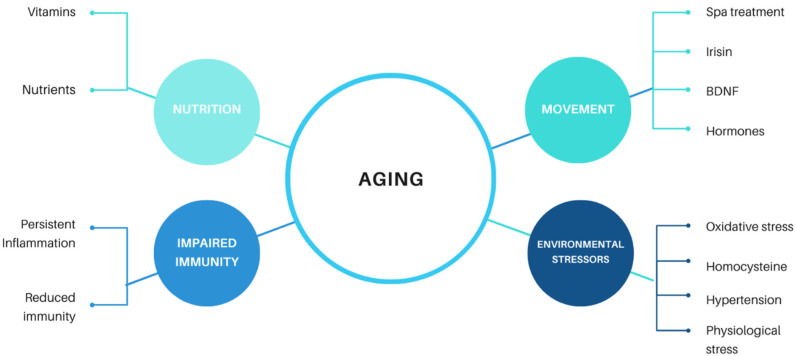
Factors influencing aging.

We can conclude that aging is a complex physiological process that has been observed across all living organisms, including the simplest organisms. It is related to a number of different both external and internal effectors (Figure 1). At a human level, we generally perceive aging as a loss of vitality, regeneration and a tendency to develop age-related diseases. Manifestations of senescence overlap and include impaired immune and endocrine system function, reduced function of organs (including the brain) and the development of neurodegenerative diseases. Although the global population is aging, it seems the proportion of human life without disease has not changed much. In the age of COVID-19, aging has become one of the most discussed topics, as older adults are the most at-risk group for COVID-19.

The research shows that regular physical activity is among the most important self-care recommendations for maintaining physical and mental wellbeing for as long as possible. Movement affects the limbic system, especially the hypothalamus. The processes between the endocrine and immune systems are activated by aerobic exercise.

Maintaining a healthy lifestyle has always been a more effective and efficient solution than medical intervention and treatment of individual diseases. The same applies to the question of how we should best care for an aging population. Overall life expectancy is not as important to a person as maintaining self-sufficiency and a high quality of life or as long as possible. In this regard, research confirms that optimizing physical mobility and mental health through movement proves to be the most recommended, simplest and cheapest option for the care of our aging populations.

## Data Availability

Excluded as the study did not report any measured data.
